# HMM_RA: An Improved Method for Alpha-Helical Transmembrane Protein Topology Prediction

**DOI:** 10.4137/bbi.s358

**Published:** 2008-01-31

**Authors:** Jing Hu, Changhui Yan

**Affiliations:** 1 Department of Computer Science, Utah State University, Logan, UT 84322 U.S.A

**Keywords:** Alpha helical transmembrane, HMM, Reduced alphabet, Topology prediction

## Abstract

α-helical transmembrane (TM) proteins play important and diverse functional roles in cells. The ability to predict the topology of these proteins is important for identifying functional sites and inferring function of membrane proteins. This paper presents a Hidden Markov Model (referred to as HMM_RA) that can predict the topology of α-helical transmembrane proteins with improved performance. HMM_RA adopts the same structure as the HMMTOP method, which has five modules: inside loop, inside helix tail, membrane helix, outside helix tail and outside loop. Each module consists of one or multiple states. HMM_RA allows using reduced alphabets to encode protein sequences. Thus, each state of HMM_RA is associated with *n* emission probabilities, where *n* is the size of the reduced alphabet set. Direct comparisons using two standard data sets show that HMM_RA consistently outperforms HMMTOP and TMHMM in topology prediction. Specifically, on a high-quality data set of 83 proteins, HMM_RA outperforms HMMTOP by up to 7.6% in topology accuracy and 6.4% in α-helices location accuracy. On the same data set, HMM_RA outperforms TMHMM by up to 6.4% in topology accuracy and 2.9% in location accuracy. Comparison also shows that HMM_RA achieves comparable performance as Phobius, a recently published method.

## Introduction

About 20%–30% of all genome sequences encode integral membrane proteins (Jones, 1998; Krogh et al. 2001; Wallin and von-Heijne, 1998). α-helical transmembrane (TM) proteins are the integral TM proteins that have α-helixes in the membrane-spanning regions. The extreme difficulties of crystallizing membrane proteins by X-ray or NMR have called for computational methods that can correctly predict the locations of TM segments and the topology of TM proteins. Because of the obvious statistical distribution of hydrophobic residues in α-helical TM segments, earlier methods identify TM segments using hydrophobicity analysis. In those methods, if the total hydrophobicity value of a fixed-length window of amino acids is greater than a user-defined threshold, it is predicted to be a TM segment (Engelman et al. 1986; von-Heijne, 1992). These methods have been improved by considering the charge and amphiphilicity distribution (Landolt-Marticorena et al. 1993; Sipos and von-Heijne, 1993). Although these methods worked well in identifying TM segments, they were not successful at predicting the topology of TM proteins.

Many methods, such as TopPred (Claros and von-Heijne, 1994), MEMSAT (Jones, 2007), PHD (Rost et al. 1996), ENSEMBLE (Martelli et al. 2003), HMMTOP (Tusnady and Simon, 1998; Tusnady and Simon, 2001), TMHMM (Krogh et al. 2001; Sonnhammer et al. 1998), PRODIV_TMHMM (Viklund and Elofsson, 2004), TMMOD (Kahsay et al. 2005), Phobius (Kall et al. 2007), THUMBUP/UMDHMM^TMHP^ (Zhou and Zhou, 2003), PONGO (Amico et al. 2006) and HMM-TM (Bagos et al. 2006), have been developed to predict the topology of α-helical TM proteins. Several studies (Chen et al. 2002; Cuthbertson et al. 2005; Kall and Sonnhammer, 2002; Melen et al. 2003; Moller et al. 2001) have evaluated and compared the reliabilities of different methods. HMMTOP and TMHMM have been consistently rated among the best methods. Both HMMTOP and TMHMM are based on hidden Markov models. Each method defines a set of states corresponding to certain regions of α-helical TM proteins. The architecture of TMHMM includes seven modules: helix core, inside cap, outside cap, cytoplasmic loop, short non-cytoplasmic loop, long non-cytoplasmic loop and globular domains (Krogh et al. 2001; Sonnhammer et al. 1998). The architecture of HMMTOP consists of five modules: inside loop, inside helix tail, membrane helix, outside helix tail and outside loop (Tusnady and Simon, 1998; Tusnady and Simon, 2001). Each module consists of one or multiple states. Each state is associated with a probability distribution over 20 amino acids. HMMTOP can make prediction in either single sequence mode or multiple sequence mode. In single mode, the topology of a protein is predicted using only the primary sequence of the protein as input. In multiple sequence mode, the topology of a protein is predicted using its sequence and its homologous sequences as input. Usually, HMMTOP can achieve better performance in multiple sequence mode. TMHMM only work in single sequence mode.

Here, we present a Hidden Markov model (referred to as HMM_RA) that can predict the topology of α-helical TM proteins with improved performance. HMM_RA adopts the same structure as HMMTOP and allows the use of reduced alphabets to represent amino acids. Each state of HMM_RA is associated with a probability distribution over *n* symbols, where *n* is the size the reduced alphabet set. Direct comparisons using two standard data sets show that HMM_RA consistently outperforms HMMTOP and TMHMM in topology prediction and α-helices location prediction. Specifically, on a high-quality data set of 83 proteins, HMM_RA outperforms TMHMM by up to 7.6% in topology accuracy and 6.4% in α-helices location accuracy. On the same data set, HMM_RA outperforms HMMTOP by up to 6.4% in topology accuracy and 2.9% in location accuracy.

## Materials and Methods

### Data sets

Two well-annotated sets of α-helical TM proteins were obtained from the TMHMM website (http://www.cbs.dtu.dk/~krogh/TMHMM/) (Krogh et al. 2001; Sonnhammer et al. 1998). The first data set (referred to as *set_*160) contains ~160 proteins, among which 108 are multiple-spanning membrane proteins and 52 are single-spanning. The second data set (referred to as *set_*83) is a subset of set_160. It contains 83 proteins (38 multi-spanning and 45 single-spanning) whose topologies have been experimentally determined.

### Cross-validations

In Sonnhammer et al. (1998), set_160 and set_83 were used to evaluate the TMHMM method using ten-fold cross-validations. In this study, ten-fold cross-validations were also used to evaluate HMM_RA and HMMTOP. The cross-validations were carried out using the same data set partition as in Sonnhammer et al. (1998) (available at http://www.cbs.dtu.dk/krogh/TMHMM/). Briefly, the data set was divided into ten even subsets. The sequence identity between any two proteins from different subsets is less than 25%. Methods were trained using nine subsets and tested using the remaining subset. This procedure was repeated ten times with each subset being used as test set once.

### Reduced alphabets of amino acids

There are 20 naturally occurred amino acids. It is well known that some amino acids share similar physicochemical features. Many studies (Chan, 1999; Fan and Wang, 2003; Li et al. 2003; Murphy et al. 2000) have clustered amino acids into groups based on different properties and used reduced alphabets to represent them. Reduced alphabets have been shown to be helpful in function and structure predictions (Francisco Melo, 2006; Murphy et al. 2000; Ogul and Mumcuoglu, 2007). In this study, we try two series of reduced alphabets developed in previous studies: One series from Murphy et al. (2000) (Table 1) and another from Li et al. (2003) (Table 2). We named each reduced alphabet set using author’s name followed by a number that denotes the size of the alphabet set, e.g. Murphy_15, Murphy_10, Murphy_8, Murphy_4, Murphy_2, Li_10, Li_9, Li_8, Li_7, Li_6, Li_4, and Li_2.

### HMM_RA

**Figure 1:**
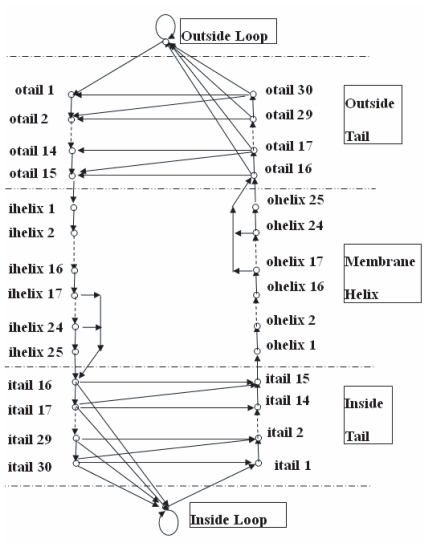
Architecture of the HMM_RA. The model has 5 modules: inside loop, inside tail, membrane helix, outside tail and outside loop. Each module consists of one or multiple states.

We modified the HMMTOP method (Tusnady and Simon, 1998) and developed a new method (referred to as HMM_RA, i.e. Hdiden Markov Model with Reduced Alphabets) that can predict the topology of α-helical TM proteins using reduced alphabets. HMM_RA has the same structure as HMMTOP (Fig. 1). The model has five modules: inside loop, inside helix tail, membrane helix, outside helix tail and outside loop. Each module consists of one or multiple states. In HMMTOP, each state is associated with 20 emission probabilities, corresponding to the 20 amino acids. In HMM_RA, each state is associated with *n* emission probabilities, where *n* is the size of the reduced alphabet used.

### Single sequence mode vs. multiple sequence mode

HMMTOP can make prediction in either single sequence mode or multiple sequence mode. In single mode, the topology of a protein is predicted using only the primary sequence of the protein as input. In multiple sequence mode, the topology of a protein is predicted using its sequence and its homologous sequences as input. HMM_RA can also run in single sequence mode and multiple sequence mode. When multiple sequence mode was chosen, the predictions were carried out as described in Tusnady and Simon (1998): the BLAST program (Altschul et al. 1997) was used to search for homologous sequences. Sequences sharing >25% identity with the query protein were selected. If more than 50 homologous sequences were found only the best 50 (including the query sequence) were used.

### Measures

One issue in the evaluation of topology prediction is the minimal overlap required between the predicted and observed helices. A minimal overlap of 3 residues has been used in most of the previous studies (Chen et al. 2002; Cuthbertson et al. 2005; Jones et al. 1994; Persson and Argos, 1996; Sonnhammer et al. 1998; von-Heijne, 1992). Moller et al. (2001) required an overlap of at least 9 residues. We tried different minimal overlaps in the range from 3 to 9. Only minor differences were observed in the prediction accuracy. More importantly, consistent results were obtained in the comparisons of HMM_RA with HMMTOP and TMHMM. Note that in the comparisons, we used the same criterion to evaluate different methods. Since a minimal overlap of 3 residues was used in most studies, in this study, we report the results with a minimal overlap of 3 residues. Thus, the location of a TM helix is correctly predicted if the overlap between the predicted helix and the true helix is at least 3. A protein’s topology is correctly predicted if the locations and directions of all its TM helices are correctly predicted. Two measures are used to evaluate the methods:

*Topology Accuracy* = *N*_T_*/N*, where *N_T_* is the number of proteins whose topology is correctly predicted and *N* is total number of proteins.

*Location accuracy = N_L_^/^N*, where *N_L_* is the number of proteins whose TM helices are all correctly localized and *N* is defined as above.

## Results

### HMM_RA performs best when Li_8, Li_9 and murphy_8 are used

**Figure 2:**
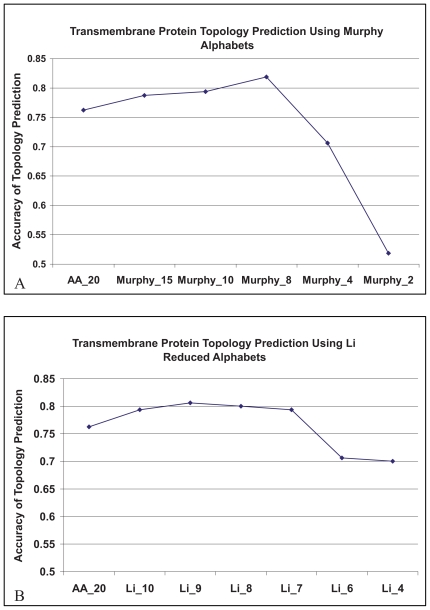
The performance of HMM_RA. **(A)** Various sets of reduced alphabets from Murphy (2000) were used to encode protein sequences; **(B)** Various sets of reduced alphabets from Li alphabets (Li et al. 2003) were used to encode protein sequences. Set_160 was used to evaluate the method using multiple sequence mode. AA_20: 20 alphabets were used to encode protein sequences. We named each reduced alphabet set using author’s name followed by a number that denotes the size of the alphabet set.

Set_160 is used to evaluate HMM_RA using multiple sequence mode. First, we encode protein sequences using the various sets of reduced alphabets developed by Murphy et al. (2000). The results (Fig. 2a) shows that as the alphabet size decreases starting from 20, the accuracy of topology predictions first increases, reaching a maximum of 81.9% when Murphy_8 is used, and then drops rapidly. We then encode protein sequences using the various sets of reduced alphabets developed by Li et al. (2003). A similar increase-then-descrease trend is observed in the accuracy of topology prediction (Fig. 2b). The results show that when reduced alphabet Li_9 is used, HMM_RA achieves the best accuracy (80.6%). When Li_8 is used HMM_SA also achieves an accuracy (80%) that is very close to the best.

### HMM_RA achieves better performance in high quality data set

**Figure 3:**
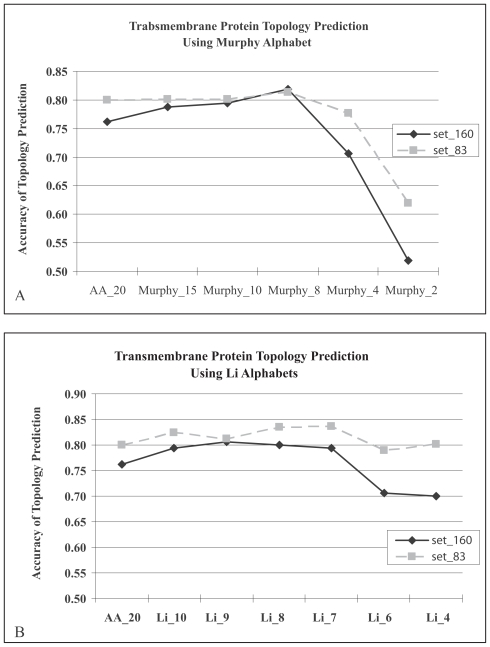
HMM_RA achieves better performance in the high-quality data set. (**A**) Various sets of reduced alphabets from Murphy (2000) were used to encode protein sequences; (**B**) Various sets of reduced alphabets from Li alphabets (Li et al. 2003) were used to encode protein sequences. Set_160 and set_83 were used to evaluate the method using multiple sequence mode. AA_20: 20 alphabets were used to encode amino acids. We named each reduced alphabet set using author’s name followed by a number that denotes the size of the alphabet set.

Set_83 is a subset of Set_160. The topology of proteins in Set_83 have been experimentally confirmed. We evaluate HMM_RA using set_83, and compare the results with those obtained using set_160. The results (Figs. 3A, 3B) show that HMM_RA achieves better performance in the high-quality data set, set_83.

### Comparisons with previously published methods

Many methods have been developed to predict the topology of α-helical membrane proteins. TMHMM (Krogh et al. 2001; Sonnhammer et al. 1998) and HMMTOP (Tusnady and Simon, 1998; Tusnady and Simon, 2001) are two best-ranking methods among them. Here, we compare HMM_RA with these two methods. HMMTOP (version 2.0) is downloaded from http://www.enzim.hu/hmmtop/. HMM_RA and HMMTOP are evaluated on set_160 and set_83 using ten-fold cross-validations as described in Materials and Methods. The cross-validations are carried out using the same data partition as in Sonnhammer et al. (1998), such that similarity between any two sequences from different subsets is less than 25%. The results for TMHMM are obtained from Sonnhammer et al. (1998). Thus, in the comparisons, the three methods are evaluated using the same training sets and test sets.

Results from previous sections show that HMM_RA can achieve one of the best results in both set_83 and set_160 when reduced alphabet Li_8 is used. Thus, in the comparisons, Li_8 is used to encode protein sequences for HMM_RA. First, we use set_83 to compare the performance of the three methods because set_83 is a high-quality data set.

The results (Table 3) show that compared with HMMTOP, HMM_RA achieves an improvement of 7.6% in topology accuracy and an improvement of 6.4% in location accuracy when single sequence mode is used. When multiple sequence mode is used, HMM_RA outperforms HMMTOP by 3.5% in topology accuracy and 2.4% in location accuracy.

TMHMM only works in single sequence mode. When single sequence mode is used for HMM_RA, HMM_RA outperforms TMHMM by 5.4% in topology accuracy and 0.7% in location accuracy. When multiple sequence mode is used for HMM_ RA, the improvement is increased to 6.4% in topology accuracy and 2.9% in location accuracy.

In additional to set_83, set_160 is also used to evaluate and compare the three methods. The results (Table 4) show that HMM_RA still outperforms TMHMM and HMMTOP on set_160.

We also compare HMM_RA with a recently published method Phobius (Kall et al. 2007). Set_160 is submitted to the Phobius server. The results show that Phobius achieves 80.0% accuracy in topology prediction. In comparison, the HMM_RA also achieves 80.0% accuracy on the same dataset. It is worth to point out that, different from the comparisons between HMM_RA, HMMTOP and TMHMM in which the same ten-fold cross-validation is used to evaluate all the methods, here, in the comparison of Phobius and HMM_RA, we have no control over the training set of Phobius. Thus, the data set that the Phobius server was trained on may have a big overlap with the test data set, *set_160*. Therefore, the accuracy of Phobius may have been overestimated. On the other hand, HMM_RA is evaluated using a stringent ten-fold cross-validation. Remarkably, HMM_RA still achieves the same accuracy as Phobius.

## Discussion

In summary, we present a method, HMM_RA, that can predict the topology of α-helical TM proteins with improved performance. Direct comparison shows that HMM_RA can outperform HMMTOP by up to 7.6% in topology accuracy and 6.4% in α-helices location accuracy and outperform TMHMM by up to 6.4% in topology accuracy and 2.9% in location accuracy.

Using reduced alphabets to encode amino acids can reduce the complexity of protein sequence. In this study, using reduced alphabets has the additional benefit of reducing the number of parameters (emission probabilities) in the models. Different amino acids can perform a similar function because they have similar physiochemical properties or they are close in the evolution. Clustering the amino acids based on these properties can produce reduced alphabets without losing information for function or structure identification. Using reduced alphabets to represent amino acids help to identify the features essential for the function. In this study, as the alphabet size decreases from 20, the performance of HMM_RA first increases, reaching a maximal value, and then decreases. Ongoing research in our group analyzes the characteristics of the reduced alphabet on which the best performance is achieved to search for physical-chemical properties that are indicative of TM locations and topology.

TMMTOP_RA work in either single sequence mode or multiple sequence mode. On both data sets used in this study, TMMTOP_RA achieves better performance when multiple sequence mode is used as input. Another factor that affects the performance is data quality. On high-quality data set, TMMTOP_RA can achieve better performance.

## Figures and Tables

**Table 1:** Reduced alphabet sets from Murphy et al. (2000).

	LV	C	A	G	S	T	P	F	W	E	D	N	Q	K	H
	IM							Y						R	
Murphy_15*	L	C	A	G	S	T	P	F	W	E	D	N	Q	K	H
Murphy_10	L	C	A	G	S		P	F		E				K	H
Murphy_8	L		A		S		P	F		E				K	H
Murphy_4	L		A					F		E					
Murphy_2	L							E							

* Each reduced alphabet set is given a name, which includes the author’s name followed by a number denoting the size of the alphabet.

**Table 2:** Reduced alphabet sets from Li et al. (2003).

	C	F	M	I	G	P	A	N	Q	R
		Y	L	V			T	H	E	K
		W					S		D	
Li_10*	C	Y	L	V	G	P	S	N	E	K
Li_9	C		L	V	G	P	S	N	E	K
Li_8	C		L		G	P	S	N	E	K
Li_7	C		L		G	P	S	N		K
Li_6	C		L		G	P	S	N		
Li_4	C		L		G			N		

* Each reduced alphabet set is given a name, which includes the author’s name followed by a number denoting the size of the alphabet.

**Table 3:** Comparisons of different methods using *set_83.*

Input mode	Method	Topology Accuracy	Location Accuracy
Single Sequence	HMM_RA (using Li_8)	**82.5%**	**83.8%**
	HMMTOP	74.9%	77.4%
	TMHMM	77.1%	83.1%
Multiple Sequences	HMM_RA (using Li_8)	**83.5%**	**86.0%**
	HMMTOP	80.0%	83.6%

**Table 4:** Comparisons of different methods using *set_160.*

Input mode	Method	Topology Accuracy	Location Accuracy
Single Sequence	HMM_RA (using Li_8)	**77.5%**	**83.8%**
	HMMTOP	75.0%	80.6%
	TMHMM	76.9%	83.8%
Multiple Sequences	HMM_RA (using Li_8)	**80.0%**	**84.4%**
	HMMTOP	76.3%	81.9%
